# Educating about radiation risks in high schools: towards improved public understanding of the complexity of low-dose radiation health effects

**DOI:** 10.1007/s00411-018-0763-4

**Published:** 2018-11-22

**Authors:** Andrzej Wojcik, Karim Hamza, Iann Lundegård, Margareta Enghag, Karin Haglund, Leena Arvanitis, Linda Schenk

**Affiliations:** 1 MBW Department, Centre for Radiation Protection Research, Stockholm University, Svante Arrhenius väg 20C, 106 91 Stockholm, Sweden; 2 Institute of Biology, Jan Kochanowski University, Kielce, Poland; 3 Department of Mathematics and Science Education, Stockholm University, Stockholm, Sweden; 4 Tumba Gymnasium, Botkyrka kommun, Stockholm, Sweden; 5 Blackeberg Gymnasium, Stockholm, Sweden; 6 Unit of Work Environment Toxicology, Institute of Environmental Medicine, Karolinska Institutet, Stockholm, Sweden; 7 Department of Philosophy and History, KTH, Royal Institute of Technology, Stockholm, Sweden

**Keywords:** Risk, Low doses, Education, Stochastic effect, Cancer

## Abstract

The levels of stochastic health effects following exposure to low doses of ionising radiation are not well known. A consequence of the uncertainty is that any radiation exposure is met with deep concern—both by the public and by scientists who disagree about how the partly conflicting results from low-dose studies should be interpreted. The concern is not limited to ionising radiation but is inherent to other areas of modern technologies such as biotechnology or electromagnetic fields. The everyday presence of advanced technologies confronts people with the necessity to take decisions and there is an ongoing debate regarding both the nature and magnitude of potential risks and how education efforts may empower peoples´ decision-making. In the field of radiation research there are different opinions regarding the optimal education methods, spanning from the idea that peoples’ fears will be eliminated by introducing dose thresholds below which the risk is assumed to be zero, to suggestions of concentrating research efforts in an attempt to eliminate all uncertainties regarding the effects of low doses. The aim of this paper was to present our approach which is based on developing an education program at the secondary school level where students learn to understand the role of science in society. Teaching about radiation risk as a socio-scientific issue is not based on presenting facts but on showing risks in a broader perspective aiming at developing students’ competency in making decisions based on informed assessment. We hope to stimulate and encourage other researchers to pursue similar approaches.

## Introduction

Health effects of ionising radiation are divided into two categories: deterministic effects (also referred to as tissue effects) and stochastic effects (ICRP 103 2007). The former, such as skin necrosis, are unavoidable but only after a certain threshold of dose was exceeded. Thus, staying below the dose threshold provides full protection. The latter, such as cancer, do not have a threshold and occur with a certain probability which is proportional to the dose. Thus, reducing the absorbed dose does not provide full protection, but only minimises the probability of the adverse effect manifesting. Moreover, large uncertainties prevail over the shape of the dose–response curve in the low-dose region, with opinions spanning between the existence of a threshold below which there is no excess risk, to the belief that, per unit dose, low doses are more detrimental than high doses (UNSCEAR 2000).

Deterministic radiation effects which can be simply avoided by keeping the radiation exposures below their threshold dose cause little public concern. With sufficient knowledge of dose–response relationships and efficient regulations, such effects can be prevented. The situation is quite different for stochastic effects which cannot be fully prevented unless the exposure is completely eliminated. Hence, the question inescapably arises as to which exposure level is acceptable. An essential element of the radiation protection system is justification, which implies that a radiation exposure is acceptable when the benefit of exposure exceeds the risk of the detriment (ICRP 103 2007). This approach is reasonable under the assumption that the dose–response relationship for the risk factor is well documented. This is not the case for cancer induced by low doses of ionising radiation (Brenner et al. 2003).

A consequence of the uncertainty in the quantification of risk of low doses, but also of the stochastic nature of risk, is that radiation exposure is often met with deep concern and even fear (Slovic 1996). Psychological effects following the two major accidents in nuclear power plants at the Three Mile Island and Fukushima Daiichi, where the public was never exposed to high-radiation doses, as well as Chernobyl, where the majority of exposed people received low doses, dramatically illustrate the situation (Bromet 2014). Another example is food preservation by ionising radiation which does not reduce the quality of food but nevertheless is prohibited in many countries because of (to all appearances unfounded) concern for adverse health effects (Heddle et al. 2014). In effect, public debates regarding radiation technologies are seldom balanced because the public perceptions of radiation risk differ from the assessment of experts (Perko 2014; Slovic 1996). This can lead to decisions, such as closing down of nuclear power plants, which have a major economic and environmental impacts because energy is then often derived from combustion of fossil fuel.

Opinions differ regarding the optimal solution to the problem. One suggested approach is to replace the currently adopted linear non-threshold theory (LNT) approach (ICRP 103 2007) by a permissible dose threshold (Sacks et al. 2016) in the belief that not only is radiation harmless below a certain threshold level, but even has positive, so-called hormetic, health effects (Hamblin 2007; Jaworowski 2010). Another approach is to intensify research efforts on low-dose health effects so that more knowledge is gained and can be passed to members of the public and to politicians who will then take evidence-based decisions (Salomaa et al. 2017). Yet another approach is to educate young scientists in the field of radiation research so that they have knowledge and understanding of the field and can wisely represent it towards the public and politicians who take decisions regarding radiation protection (Smith et al. 2017). In short, evidence-based and honest public education about radiation effects at low doses will result in a more balanced perception of risk. However, the problem is that scientists strongly disagree among themselves about such fundamental issues as the shape of the dose–response relationship in the low-dose range and the magnitude of risk per unit dose (Wakeford 2008). Indeed, it appears that there is no simple relationship between increased knowledge of facts and “better” decisions (Perko et al. 2012).

The mechanisms of how people make decisions on risk issues are very complex (Adams 2000) and there is vivid debate regarding the question how socio-scientific issues, such as risks associated with modern technologies, may be taught already at secondary school level (Christensen 2009). Indeed, complex socio-scientific issues such as health risks associated with advanced technologies are appreciated within the science education field as means to develop teaching that promotes students’ decision-making skills, illustrates the fallible nature of science, and advances the understanding of the role of science in society. Socio-scientific issues require a teaching that is not based on only presenting facts, but rather on showing risks in a broader perspective aiming at developing students’ competency in making decisions based on informed assessment of a complex state of the art (Roberts 2007; Zeidler et al. 2005). Risks associated with modern technologies include not only ionising radiation from nuclear power plants, industry and medicine but also, for example, the rapidly growing field of biotechnology. Moreover, risk-related socio-scientific issues offer students an opportunity to become familiar with the concept of risk being a multifaceted term that is impossible to define in a simple way (Schenk et al. 2018a).

The aim of this paper was to present our experiences from the 3-year research project RiskEdu (http://www.riskedu.se), which is a collaboration between science education researchers, science teachers, and researchers in risk assessment and radiation risk management. We received national funding to investigate how science teaching can support the development of high-school students’ competency in making decisions based on informed risk assessment in societal issues involving exposure to threats associated with modern technologies, such as ionizing radiation or the rapidly growing number of applications of biotechnology. Here we give an overview of the project, with focus on radiation risks, and hope to stimulate and encourage other researchers to pursue similar approaches.

## The project RISKEDU

The project RISKEDU is a collaborative effort of three researchers in the field of science education (Karim Hamza—the project coordinator, Margareta Enghag and Iann Lundegård): one in the field of toxicology and risk (Linda Schenk), one in the field of radiation biology and radiation protection (Andrzej Wojcik) and two high school teachers (Leena Arvanitis who teaches biology and natural science and Karin Haglund who teaches physics and chemistry). The collaboration consists of repeated cycles of planning–teaching–analysis. In each cycle, planning and analysis are carried out jointly by teachers and researchers, whereas actual teaching is done primarily by the teachers, the researchers being responsible for documenting the teaching activities. Informed consent was collected from all students and additional teachers that participated in the research reviewed herein. Short examples of teaching cycles are given below, but in order to justify our approach, we would first like to describe the different approaches to define risk and discuss teaching some of its aspects in the age of risk society.

## Defining risk

We encounter risk every day in our lives, and make numerous risk related decisions—risk–risk or risk–benefit trade-offs—mostly without much deliberate reflection on the matter. However, we stumble when faced with the request of defining it. Interestingly, there is no widely accepted definition of the term “risk” (Adams 2000). This is not due to lack of efforts. Indeed, within the risk management and decision analysis literature many authors have attempted to provide definitions of the concept, or defend a particular definition or operationalisation of risk for risk assessment (Aven and Reniers 2013). It has been recognised that risk as a concept cannot be properly captured by a single operationalisation, rather any operationalisation has to be adapted to the decision-making context (Risk glossary, retrieved from: http://www.sra.org/resources).

There are a number of definitions in which the uncertain nature of risk is operationalized as probability (not discussed herein but see (Aven and Reniers 2013) for a definition of probability). An early attempt to assign a definition to the term risk that is tied to the ability of its measurement was made by the economist Knight in 1921. Focusing on the situation of incomplete knowledge, Knight wished to distinguish between measureable and unmeasurable uncertainty. He assigned the term risk to the former kind and uncertainty to the latter (Knight 1921). In 1983, a UK Royal Society working group defined risk as “the probability that a particular adverse event occurs during a stated period of time, or results from a particular challenge” (Royal Society 1983). Risk as probability is currently a common understanding in medicine and associated disciplines. In epidemiology as well as in radiation research, the term risk is generally used to designate the probability to contract a certain disorder, such as cancer or non-cancer disease. However, we can also argue that the severity of the disease, or the investigated health outcome, plays a major role for the understanding of risk in this context. For instance, a relative risk of, say, 5 for the poorly curable lung cancer has a different value than the relative risk of 5 for the well-curable thyroid cancer. This type of risk understanding can be better described as the combined outcome of probability and consequence.

The above considerations are also to some degree reflected in the definition of risk as being the statistical expectation value of unwanted events which may or may not occur (Hansson 2004). Risk as an expectation value denotes a numerical representation of risk obtained by multiplying the probability of an unwanted event with a measure of its negative value. It can be noted that in the UK Royal Academy 1983 report on risk, this compound measure combining the probability and magnitude of an adverse effect was defined as “detriment” (Adams 2000; Royal Society 1983). Risk analyses of industrial processes often express risk/detriment as expected numbers of fatalities per year of operation or expected monetary losses. In health risk assessment of hazardous exposures this definition is sometimes rewritten to risk = exposure × consequences: implicitly an increased exposure to the hazardous agent increases the probability of an adverse health effect occurring.

However, defining risk as the expectation value overly simplifies the situation as it assigns a single numerical value to risk. This may equate a scenario of low harm and high probability to one with a severe harm and low probability (Kaplan and Garrick 1981). These insights triggered the call for more complex definitions such as Kaplan and Garrick’s risk as a set of triplets of scenario, probability and consequence, covering all possible (according to the best available knowledge) scenarios. In other words, Kaplan and Garrick argue that risk should capture the following questions: What can happen? How likely is that to happen? If it does happen, what are the consequences? (Kaplan and Garrick 1981).

On top of the disparate definitions from the specialised realm, we also have the situation where the word risk is used in a non-specialised sense. As within the different specialised uses of risk, there are many different meanings of risk in our everyday language. The polysemy, that is the coexistence of many possible meanings, of the noun “risk” can be exemplified with the following ten answers from school students (16–17 years of age) in our RISKEDU survey to the question: “what do you think of when you hear the word risk”?:

- That there is something unwanted or dangerous with an event.
- Danger and uncertainty.
- Dangerous events that may happen.
- You may lose something.
- Something that is dangerous and can cause harm.
- I am thinking about causes of something becoming exposed to danger.
- Risk of getting a disease later in life, such as cancer.
- The probability that something unwanted will happen.

In these sentences we see meanings such as risk being an unwanted event (consequence), the potential for an unwanted event, the cause of an unwanted event, or the probability for an unwanted event compare to Hansson (2004).

Boholm et al. (2016) discuss the polysemy of risk from a risk communication perspective and suggest that the differences between its every day and technical uses may give rise to misunderstandings that could hamper communication efforts. Different understandings of the word “risk” have also been identified as one potential cause behind the often-found disparities between laypersons’ and experts’ risk perception (Perko 2014; Sjöberg 1999a, b; Slovic 2016). Leaning on these observations we have argued that pre-university education may be a good place to address different ways to conceptualise risk (Schenk et al. 2018a). The science classroom may be where the students first encounter the specialist uses of “risk” and the contrast to their own every-day understanding of the term. However, rather than just the polysemy of the word risk, it is the complexity that this polysemy reflects that is of main interest for science educators. In the next section we will shortly illustrate not only some of the challenges, but also opportunities of teaching about risk in the age of risk society.

## Teaching about risk in the age of the risk society

According to the sociologist Ulrich Beck (Beck 1986), a major problem of the modern, technologically advanced society is exposure of its members to global risks which originate from human activity. The advanced degree and complexity of technologies, as well as the often invisible nature of threats, make them difficult to perceive and understand. Environmental contamination with ionising radiation is a good example. In order to appreciate the extent and impact of threats, members of the public must rely on experts, who usually are scientists, and on media. Self-acquired knowledge and the traditional wisdom of the elderly are of little help. But, as outlined in the introduction, scientists often disagree and publish contradictory assessments of the degree of a problem and make different predictions regarding the effect that the threats may have on the environment and on human health. This situation, which is an inherent trait of scientific research, leaves the public in a state of confusion and may provoke distrust, in particular, if scientists or politicians make mistakes when assessing and managing the potential threats associated with new technologies. A much quoted example is the UK’s mad cow disease crisis of 1996, where the governmental institutions declared there to be no risk before the science was in; the matter worsened when no-risk message was continued also after contradictory evidence had been published (Jasanoff 1997; Leiss and Powell 2002). An example from the radiation research field is the wrong prediction regarding behaviour of ^137^Cs in Wales and Cumbria following the Chernobyl accident in 1986. Based on results of previous experiments, scientists predicted that the impact on livestock of ^137^Cs, which rained down following the accident, would be negligible because the radionuclide would be bound by the soil. This prediction was wrong, as the isotope was taken up by grass and subsequently eaten by the grazing sheep leading to contamination levels above the permissible limits (Wynne 1992). The public may perceive such mistakes as evidence for ignorance or even malevolence driven by hidden desires to draw benefit from hiding the truth. Although the option of malevolence can never be excluded, there is no doubt that the major cause of disagreement among scientists and mistakes is the high complexity of technologies and risks and the limitations of experimental tools to foresee all threats and define the level of risk. Results from epidemiological studies on health risks after low-dose exposure are haunted by uncertainty due to problems with statistical power (Brenner et al. 2003). Consequently, levels of risk considered acceptable are associated with radiation doses which cause health effects that are scientifically undetectable [dose limit of 1 mSv per year for the general population (ICRP 103 2007)]. What exactly is considered acceptable is an ethical decision, based on values such as prudence. This is true not only in the field of radiological protection but also in protection against many hazardous chemicals (Hansson 2002). In situations like this regulations rely on the ethical principle of prudence (ICRP 138 2018). However, how do we know that the regulations are still safe enough? The discussion is far from being academic, because the society must take decisions with respect to such issues as the design and location of spent fuel repository, where the potential impact on the environment of storing radioactive waste is based on predictions (Kautsky et al. 2013).

How should the society be educated so that it takes the right decisions? By providing information about the objective levels of risk? These are not known due to uncertainties associated with results from scientific investigations. Moreover, how should the problem of unknown unknowns (Alles 2009) be explained?

The optimal approach appears to be to educate scientifically literate citizens, preferably already at the school level (Ratcliffe et al. 2005). The concept of scientific literacy has evolved from the simplified idea that knowledge of basic science concepts will be sufficient for people to take informed decisions, to the realization that scientifically literate citizens require several other competencies as well, for instance concerning the nature of scientific knowledge, how to judge between contradictory knowledge claims, and how to integrate knowledge and value claims in issues involving the need to make formal or informal risk assessments (Liu 2009; Roberts 2007). In the current project, a guiding principle is to accomplish this complex educational challenge by giving school students tasks which require taking decisions in problem situations where the process of decision making involves weighing of pros and cons. In this way, students are allowed to experience (in the sense of the Swedish word “genomleva” meaning live through) situations where no simple solutions exist, but where a decision nevertheless must be taken. The idea is that the more students deal with this kind of complex problems that socio-scientific issues pose (Sadler and Donelly 2006), the better they will be off when facing such issues later in life. Examples of such tasks are given below.

## Examples of teaching cycles

The development of teaching cycles is based on educational aims of upper secondary school sciences, which include aspects such as understanding of societal relevance and communicative skills. For instance, the aims for physics state that the teaching is intended to develop students’:

1. Knowledge of the concepts, models, theories and methods of physics and understanding of how they develop.
2. Ability to analyze and seek answers to topic-related questions as well as identify, formulate and solve problems.
3. Ability to reflect on and evaluate selected strategies, methods and results.
4. Ability to plan, implement, interpret and report experiments and observations as well as ability to handle materials and equipment.
5. Knowledge of the importance of physics for individuals and society.
6. Ability to use physics skills to communicate as well as to review and use information (Swedish School National Agency of Education, 2018).

A common experience of physics teachers is the difficulty in designing and performing teaching that targets aims 5 and 6. In line with previous research in science education, we used socio-scientific issues to target these skills, and specifically risk assessment as a bridge between physics and socio-scientific issues. Risk is an important aspect of scientific literacy for acquiring skills in decision making in socio-scientific issues and there is a need for teaching scientific inquiry based on both facts and values (Kolstø 2006; Lee and Brown 2018; Sadler et al. 2007).

### Irradiation of strawberries

Irradiation of fresh food products for the purpose of preserving their shelf life is controversial (Fan 2003; Heddle et al. 2014; Ramirez-Cahero and Valdivia-Lopez 2018) and prohibited in Sweden. So is the import of irradiated food, with the exception of spices. Is this decision based on scientific evidence demonstrating an unacceptable effect of radiation on food quality? Do the food products become radioactive? Why is irradiation of food allowed in Belgium? These questions were discussed with school students using strawberries as an example. They were asked to search for arguments justifying the prohibition in Sweden. What alternative methods exist to preserve shelf life? Are they safe? Or is it better not to preserve products and take the risk of bacterial poisoning? The interventions started with a 1-h discussion about risk assessment in general. This was followed by work in groups during two–three lessons and finalised by a second general discussion. This general teaching outline has been implemented twice in the upper secondary school physics, within the module on ionizing radiation, most recently in three classes in parallel involving three different teachers.

This design-based study was based on a strategy to teach risk issues in physics, following a pragmatic and sociocultural approach (Christensen 2009; Schenk et al. 2018b; Lee and Brown 2018; Wickman et al. 2018). The lessons were planned iteratively between the lead researcher and lead teacher to reach a positive and reasonable level for students formulating risk assessments both in discussions and in writing (Eljkelhof 1986). To assist the teaching, we supplied two works on food safety and food quality of irradiated strawberries (Filho et al. 2014; Nilson and Cerda 1993) and for the second cycle we also prepared study questions to these and, for the one in English, a Swedish summary. To analyse the teaching and students’ verbal communication, lessons were video recorded (Eljkelhof 1986; Enghag et al. 2017, 2016; Kolstø 2006; Sadler et al. 2007). In addition, for closer analysis of knowledge and skill outcomes, students’ written assignments were analysed thematically (Braun and Clarke 2006).

As mentioned, this teaching cycle has been performed twice. In our first cycle our analysis showed that students did appreciate the lack of given answers to the questions under discussion. The students had no or little difficulties in forming and voicing an opinion on the matter of irradiation of strawberries. Use and citation of facts and references for these facts were, however, challenging. In this first iteration, we concluded that students needed more structure and facilitated access to relevant literature (Enghag et al. 2016). In the second cycle, 36 written group assignments were handed in across the three classes. A striking finding was that all groups were in favour of permitting irradiation of strawberries. Two-thirds of the groups made an explicit, quantitative or qualitative, assessment of the risk and the acceptability of it. We not only found that students use our recommended references in a satisfactory manner, but also that they use other references and connect to other areas than food safety and food quality, e.g., environmental issues concerning food-waste and use of pesticides. Thus, we see that students not only use facts, but also values from other areas connecting to other school subjects such as biology, chemistry as well as from their daily lives. In our continued analyses, we will have to investigate how references to different types of knowledge areas help students formulate different ideas in their risk assessment.

### Screening for thyroid cancer in children living in the Fukushima prefecture

The Fukushima Daiichi nuclear power plant disaster left many parents living in the Fukushima prefecture in a state of turmoil regarding the risk of thyroid cancer in their children. Although experts predicted that no detectable increase of thyroid cancer incidence was expected due to the low levels of doses (WHO 2012, 2013), the government of the Fukushima prefecture decided to introduce a prefecture-wide screening action to detect early stages of cancer. The outcome was dramatic in that it revealed a strong increase in cancer incidence which was interpreted by some experts as evidence for stronger than hitherto assumed carcinogenic potential of radiation (Tsuda et al. 2016), while others claimed that it is due to screening (Wakeford et al. 2016; Williams 2015). In any case, the action left the parents of positively diagnosed children with the burning question of what to do next. The events coincided with a debate about the expedience of a nationwide screening program for several cancer types, including thyroid cancer, which was introduced in South Korea in 1999 (Ahn et al. 2014). The program resulted in a dramatic increase in the incidence of thyroid cancer which was mirrored by an equivalent increase in the number of surgical interventions, leaving the mortality rates unchanged (Ahn et al. 2014). More recent voices strongly recommend to drop the program (Ahn and Welch 2015) because it leads to overdiagnosis of cancer (Welch and Black 2010). A relevant question is thus: to screen or not to screen?

Based on the above issue, we have developed teaching material that is divided into two parts and is meant as a basis for student deliberation (Arvanitis et al. 2018). The first part focuses on the question of expedience of medical screening, whereby the advantages of screening include improved curability of cancer when detected at early stages and the disadvantages include the problem of overdiagnosis. The second part focuses on Fukushima and here the students deliberate over the expedience of introducing a screening program following exposure to radiation doses, which, according to expert judgement, are not associated with a detectable increase in the incidence of thyroid cancer.

The implementation of this teaching material has not yet started, so we do not yet have any experience that could be reported.

## Discussion and conclusions

The aim of this publication was to introduce the concept of teaching high-school students about health risks associated with radiation exposure in the broader perspective of contemporary risk society (Beck 1986). We claim that the weight of education should not be placed on students solely acquiring knowledge about the scientifically established levels of health risks but rather on “living through” situations were decision taking involves weighing of risks associated with choosing one of several available options. Examples are the use of radiation for preserving strawberries or screening for thyroid cancer among people living in the contaminated areas of the Fukushima prefecture. The advantage of this teaching strategy is that school students become familiar with real-life situations which require decision taking even if objective reasons for choosing one or the other alternative do not exist. In the field of radiological protection such situations are encountered when dealing with radiation doses which cause health effects that are scientifically undetectable. Students should learn to understand that in many cases it will be their responsibility to take an informed decision. At the same time, their scientific literacy is increased because they practice interpretation of scientific data and learn about the nature of science and elements of epistemology, such as that the result of a single publication seldom reflects the objective truth.

It may seem that this approach favours the dangerous trend of shifting responsibilities from decisions makers to members of the public. However, taking own informed decisions in complex situations is an inherent element of the contemporary, technologically advanced society. Arnoldi (2009) illustrates this by describing a shift in the relationship between medical doctors and postmenopausal patients who face the decision of undergoing hormone replacement therapy (HRT). HRT relieves the troublesome menopause symptoms and decreases the risk of osteoporosis. However, this comes at the costs of increased risk of breast cancer, heart disease, and blood clots in the lungs. In the early days of HRT treatment, when the side effects of HRT were not fully realised, doctors would authoritatively advice the patient to take HRT or not. Today, in view of the side effects and ill-defined genetic factors which may influence patients´ susceptibility, doctors present the pros and cons but leave the decision with the patient. It is worth noting that the involvement of stakeholders in decision-making is in line with the ethically justified procedural value of inclusiveness which is promoted by the ICRP (ICRP 138 2018). With respect to the RISKEDU project, it can be expected that the chosen education strategy promotes students’ readiness to, as citizens, participate in the process of decision making.
